# Association of environmental chemicals & estrogen metabolites in children

**DOI:** 10.1186/s12902-015-0079-1

**Published:** 2015-12-17

**Authors:** Erin Speiser Ihde, Ji Meng Loh, Lawrence Rosen

**Affiliations:** The Deirdre Imus Environmental Health Center®, Hackensack University Medical Center, 30 Prospect Ave, Research Building, Hackensack, NJ 07601 USA; Department of Mathematical Sciences, NJ Institute of Technology, University Heights, Newark, NJ 07102 USA

**Keywords:** BPA, Bisphenol A, Phthalates, 4-nonylphenol, Parabens, Triclosan, Pediatric, Puberty, Endocrine disruption, Estrogen

## Abstract

**Background:**

The prevalence of pediatric hormonal disorders and hormonally-sensitive cancers are rising. Chemicals including bisphenol A (BPA), phthalates, parabens, 4-nonylphenol (4NP) and triclosan have been linked to disruption of endocrine pathways and altered hormonal status in both animal and human studies. Additionally, changes in estrogen metabolism have been associated with pediatric endocrine disorders and linked to estrogen-dependent cancers. The main objective of the study was to measure the presence of these environmental chemicals in prepubescent children and assess the relationship between chemical metabolites and estrogen metabolism.

**Methods:**

50 subjects (25 male, 25 female) were recruited from the principal investigator’s existing patient population at his pediatric primary care office. The first 5 boys and 5 girls in each age group (4 through 8 years old inclusive) who presented for annual examinations were included, as long as they were Tanner Stage I (prepubertal) on physical exam, without diagnosis of hormonally-related condition and/or cancer and able to give a urine sample. Urine samples were collected in glass containers for analysis of chemical and estrogen metabolites. Study kits and lab analysis were provided by Genova Diagnostics (Duluth, GA).

Summary statistics for the concentrations of each chemical metabolite as well as estrogen metabolites were computed (minimum, maximum, median and inter-quartile range) for males only, for females only and for all subjects. Comparisons between groups (e.g. males v. females) were assessed using the nonparametric Wilcoxon test, since the data was skewed. The correlation between concentrations of chemical metabolites and estrogen metabolites in prepubescent children were examined by the Spearman’s correlation coefficient (*ρ*).

**Results:**

100 % of subjects had detectable levels of at least one chemical in their urine, and 74 % had detectable levels of eight or more chemicals. 28 % of subjects had measurable levels of 4NP. No associations were found between the urine levels of chemicals and estrogen metabolites.

**Conclusions:**

Endocrine disrupting environmental chemicals were detected in all children in the study, with measurable levels of 4NP in nearly 1/3 of subjects. This is the first known published study of 4NP levels in American children. No associations were found between the urine levels of chemicals tested and estrogen metabolites. The presence of multiple chemicals in a majority of children’s urine coupled with increasing prevalence of pediatric hormonal disorders warrants further research to elucidate potential causal mechanisms in pre- and post-pubertal children.

## Background

In the United States, cancer is the leading cause of disease-related death among children and adolescents [1]. The incidence of pediatric cancers has increased at an annual rate of 0.6 % since 1975 [2]. The largest contributor to this rise has been the germ cell, trophoblastic, and other gonadal tumor category, specifically testicular and ovarian germ cell tumors [2]. Additionally, studies have concluded that the incidence of altered pubertal onset is increasing, especially in girls [3]. Both developments have been linked to environmental factors, including exposure to endocrine disrupting chemicals [4–6]. Childhood exposure to these chemicals is widespread [4]. Of greatest concern are chemicals with potential for both endocrine disruption and carcinogenesis, including bisphenol A (BPA), phthalates, parabens, 4-nonylphenol (4NP) and triclosan [5–7].

BPA is commonly found in polycarbonate plastic products including baby bottles, water bottles, food containers, in the linings of metal food cans and in dental sealants and composites [8]. BPA has been found in over 90 % of the U.S. population age six and over, with highest concentrations in children ages 6–11 [8]. Triclosan is commonly found in antibacterial hand soaps, toothpastes and household cleaning supplies [9]. Phthalate chemicals “soften” plastics to make them pliable. They are also found in personal care products, food, plastic toys and household dust [10]. Parabens are found in common personal care products including cosmetics and antiperspirants [10]. 4NP is one of the most common alkylphenols (APs) in industry, used in the production of cleaning products, plastics, rubber and personal care products, including hair products and spermicides [11]. APs are commonly used to make alkylphenolethoxylates (APEs), which are widely used surfactants and detergents [12, 13]. Despite widespread use, exposure data on 4NP is scarce. To our knowledge, there is no published data on 4NP exposures in American children [12, 14–20]. All of these five chemicals have been detected in a majority of the U.S. population [10, 12, 21].

BPA, phthalates, triclosan, parabens and 4NP have been linked to disruption of endocrine pathways and altered hormonal status in both animal and human studies. Two pediatric studies have linked phthalate exposure to precocious puberty in girls [6, 13] while another study found an association between urinary concentrations of high-molecular weight phthalate metabolites and later pubarche [22]. BPA, triclosan, 4NP and parabens have also been associated with endocrine disruption or hormonally derived cancers in vitro and in animal models [7, 23–27]. Changes in estrogen metabolism, specifically in the ratio of 16a-Hydroxyestrone (16a-OHE1) to 2-Hydroxyestrone (2-OHE1) metabolites, have been associated with hormone disruption and linked to estrogen-dependent cancers [28, 29].

Given the widespread exposure of humans to these chemicals and the concern about environmental factors causing endocrine disruption and cancer in children, our study team investigated whether the levels of urinary chemical metabolites in children are correlated with levels and ratios of urinary estrogen metabolites. Such an association may increase the risk of these children to develop endocrine disruption, altered pubertal onset, and hormonally-associated cancers.

## Methods

Potential subjects were recruited from the principal investigator’s existing patient population at his pediatric primary care office, the Whole Child Center in Oradell, NJ from December 2012 through April 2014. Patients who come to the principal investigator’s office for a scheduled checkup were evaluated for eligibility. Subject selection was based on consecutive patients meeting the study criteria. There were 5 boys and 5 girls in each age group, 4–8 years inclusive in the study, meeting inclusion criteria as Tanner Stage 1 (displaying no secondary sexual characteristics on physical exam verified by PI), toilet trained and able to provide a urine sample. Subjects could have no diagnosed hormonally related disorder or cancer and could not be taking any hormonally active medication. At least one parent/guardian needed to be able and willing to give written informed consent.

### Recruitment procedures & informed consent

Subjects were recruited by the PI (LR), who discussed the study with parents of eligible children. As the study’s PI, Dr. Rosen is familiar with the protocol and has received documented education in the protection of human research subjects.

To comply with best practices in ethical approval and consent, this research study was conducted in accordance with the Declaration of Helsinki and was approved prior to the commencement by the HackensackUMC Institutional Review Board (HIRB protocol number Pro00003454). The protocol and de-identified clinical dataset will be made available upon request to any scientist wishing to use them for non-commercial purposes.

After having all questions answered to their satisfaction, parent(s)/guardian(s) who agreed to their child’s participation in the study were asked to sign a current, IRB approved informed consent document and HIPAA authorization form. The signed consent document included a statement informing parents/guardians that their child’s identity will remain confidential if the results of the trial are published. Pediatric assent was not obtained from subjects prior to participation.

### Lab analysis procedure

Study kits and lab analysis were provided by Genova Diagnostics (Duluth, GA). Samples were collected at the primary care office with the assistance of a parent/guardian. The urine sample was collected directly into a glass jar. Approximately 10 ml of urine was liquated from the black-capped glass collection container into the yellow-capped polypropylene plastic vial using the enclosed polyethylene pipette. Room-temperature urine was aliquot immediately into the vial upon the subject’s parent or guardian giving the glass collection jar to the research nurse. Both the black-capped collection container and the yellow-capped plastic vial were placed in a biohazard bag with a completed requisition form. Samples were refrigerated immediately after processing until shipment. Samples were shipped overnight in the specimen collection kit box with the frozen ice packs provided to Genova Diagnostics for analysis. The lab used urinary creatinine as a control for concentration and considered samples valid for analysis using creatinine. Limits of detection for the chemicals tested are included in Table 1. Our team confirmed that first morning urine was not necessary for the variables being measured in this study. The following analyses were performed on each sample:

**Table 1:** Urinary Chemicals – including and excluding measurements below the limit of detection

A = all			Includes subjects below limits of detection (replaced with LOD/2)				Excludes subjects below limits of detection			*P* values for Wilcoxon Test for males v. females
M = males only										
F = females only										
50 Subjects	# below limit of detection	% below limit of detection	Min	Median	IQR	Max	Min	Median	IQR	
BisphenolA	35	70	A = 0.05	A = 1.12	A = 2.83	A = 10.80	A = 0.17	A = 2.05	A = 2.2300	0.096
LOD = 0.101 ng/mL			M = 0.05	M = 0.58	M = 1.78	M = 10.80	M = 0.40	M = 1.61	M = 2.715	
			F = 0.05	F = 1.64	F = 2.12	F = 8.63	F = 0.17	F = 2.35	F = 1.955	
Triclosan LOD = 0.399 ng/mL	47	94	A = 0.20	A = 18.50	A = 112.75	A = 2207.00	A = 1.00	A = 21.00	A = 129.5000	0.32
			M = 0.20	M = 22.00	M = 73.00	M = 2207.00	M = 1.00	M = 24.00	M = 83.000	
			F = 0.20	F = 14.00	F = 142.00	F = 707.00	F = 1.00	F = 14.00	F = 187.500	
4-Nonylphenol	14	28	A = 0.11	A = 0.11	A = 0.13	A = 6.25	A = 0.21	A = 0.56	A = 1.5375	0.92
LOD = 0.225 ng/mL			M = 0.11	M = 0.11	M = 0.10	M = 6.25	M = 0.21	M = 0.68	M = 1.885	
			F = 0.11	F = 0.11	F = 0.14	F = 4.12	F = 0.25	F = 0.37	F = 0.865	
MEHHP	50	100	A = 7.00	A = 25.00	A = 23.50	A = 187.00	A = 7.00	A = 25.00	A = 23.5000	0.95
LOD = 1.022 μg/L			M = 8.00	M = 25.00	M = 15.00	M = 187.00	M = 8.00	M = 25.00	M = 15.000	
			F = 7.00	F = 22.00	F = 25.00	F = 104.00	F = 7.00	F = 22.00	F = 25.000	
MEHP	50	100	A = 0.40	A = 3.90	A = 3.83	A = 24.70	A = 0.40	A = 3.90	A = 3.8250	0.56
LOD = 0.199 μg/L			M = 1.00	M = 3.80	M = 3.60	M = 24.70	M = 1.00	M = 3.80	M = 3.600	
			F = 0.40	F = 4.00	F = 4.00	F = 15.20	F = 0.40	F = 4.00	F = 4.000	
MEOHP	49	98	A = 0.84	A = 13.00	A = 10.00	A = 76.00	A = 4.00	A = 13.00	A = 10.0000	0.63
LOD = 1.674 μg/L			M = 0.84	M = 13.00	M = 9.00	M = 76.00	M = 5.00	M = 13.50	M = 9.000	
			F = 4.00	F = 12.00	F = 13.00	F = 67.00	F = 4.00	F = 12.00	F = 13.000	
MetP	50	100	A = 5.00	A = 20.50	A = 14.25	A = 358.00	A = 5.00	A = 20.50	A = 14.2500	0.28
LOD = 0.830 μg/L			M = 5.00	M = 18.00	M = 8.00	M = 358.00	M = 5.00	M = 18.00	M = 8.000	
			F = 5.00	F = 22.00	F = 14.00	F = 162.00	F = 5.00	F = 22.00	F = 14.000	
Butylparaben	6	12	A = 0.17	A = 0.17	A = 0.00	A = 5.90	A = 0.60	A = 1.15	A = 1.9250	0.077
LOD = 0.332 μg/L			M = 0.17	M = 0.17	M = 0.00	M = 0.60	M = 0.60	M = 0.60	M = 0.000	
			F = 0.17	F = 0.17	F = 0.00	F = 5.90	F = 0.60	F = 1.40	F = 2.100	
Ethylparaben	36	72	A = 0.15	A = 0.60	A = 1.05	A = 8.80	A = 0.30	A = 0.90	A = 0.8000	0.63
LOD = 0.298 μg/L			M = 0.15	M = 0.50	M = 0.95	M = 1.90	M = 0.30	M = 0.90	M = 0.775	
			F = 0.15	F = 0.60	F = 1.05	F = 8.80	F = 0.40	F = 0.85	F = 0.775	
Methylparaben	50	100	A = 1.00	A = 16.50	A = 24.50	A = 1000.00	A = 1.00	A = 16.50	A = 24.5000	0.50
LOD = 0.494 μg/L			M = 1.00	M = 9.00	M = 25.00	M = 1000.00	M = 1.00	M = 9.00	M = 25.000	
			F = 2.00	F = 19.00	F = 22.00	F = 267.00	F = 2.00	F = 19.00	F = 22.000	
Propylparaben	15	30	A = 1.90	A = 1.90	A = 3.10	A = 124.00	A = 5.00	A = 8.00	A = 6.5000	0.95
LOD = 3.802 μg/L			M = 1.90	M = 1.90	M = 3.10	M = 124.00	M = 5.00	M = 9.00	M = 31.000	
			F = 1.90	F = 1.90	F = 3.10	F = 42.00	F = 5.00	F = 7.50	F = 6.000	

Note: *p* values are for tests with those < LOD replaced by LOD/2

1. Bisphenol A Profile (analysis method: Gas Chromatography/Mass Spectrometry) – Urine: includes Bisphenol A (BPA), 4-Nonylphenol (4NP), and Triclosan
2. Phthalates and Parabens (analysis method: Liquid Chromatography Tandem Mass Spectrometry) – Urine: includes metabolites of Di-2-ethylhexyl phthalate (DEHP) which include Mono-(2-ethyl-5-hydroxyhexyl) phthalate (MEHHP), Mono-2-ethylhexyl phthalate (MEHP), Mono-(2-ethyl-5-oxohexyl) phthalate (MEOHP), and Mono-ethyl phthalate (MEtP) and Parabens (Butylparaben, Ethylparaben, Methylparaben, and Propylparaben)

Estronex Estrogen Metabolites (analysis method: Ultra Performance Liquid Chromatography with Tandem Mass Spectrometry) – Urine: 2-Hydroxyestrone (2-OHE1), 2-Hydroxyestradiol (2-OHE2), 2-Hydroxyestrogens (2-OHE1 + 2) = Sum of 2-Hydroxyestrone (2-OHE1) and 2-Hydroxyestradiol (2-OHE2), 2-Methoxyestrone (2-OMeE1), 4-Hydroxyestrone (4-OHE1), 4-Methoxyestrone (4-OMeE1), and 16 alpha-hydroxyestrone (16a-OHE1).

### Statistical considerations

A total of 50 children (25 male and 25 female) between 4 and 8 years inclusive were enrolled into the study, with 5 males and 5 females for each age. Additional subjects, selected using the same recruitment procedures, were needed to account for damaged specimen collection or incomplete results due to insufficient urine collected and/or inability of the lab to result all analytes in specific cases. This resulted in a total of 68 subjects enrolled, yielding 50 evaluable subjects. Descriptive statistics and tests were conducted for each year, totaling 5 groups. Results are reported by males only, females only and both genders combined.

For exploratory purposes, summary statistics for the concentrations of each chemical metabolite were computed (minimum, maximum, median and inter-quartile range) for males, for females and for all subjects. These statistics were computed both including and excluding subjects with chemicals below the limits of detection (LOD). When computing summaries and tests with the full data, half the values of the limit of detection were used for these subjects. Table 1 shows these two sets of summary statistics. The proportion of subjects with concentrations below the LOD was calculated for each chemical metabolite. Similar summary statistics were computed for the estrogen metabolites as well.

Comparisons between groups (e.g. males v. females) were assessed using the nonparametric Wilcoxon test, since the data was skewed. The correlation between concentrations of chemical metabolites and estrogen metabolites in prepubescent children were examined by the Spearman’s correlation coefficient (*ρ*). We considered *ρ* values of 0.60-0.79 to indicate high correlation, values of 0.40-0.59 moderate, and values of 0.20-0.39 weak or low correlations. In cases where there are outliers, these were removed and the correlation coefficient recomputed to assess the effect of these outliers. All data analysis in this study was performed using R and SAS 9.2 (SAS Institute Inc., Cary, NC). Note that we do not formally account for multiple comparisons and report only the unadjusted p values. The Bonferroni correction is a simple way to correct for multiple comparisons (albeit conservatively). This can be done by the reader by reducing his/her threshold for a significant *p* value. For example, in this paper, we use a *p* value of 0.1. With multiple testing this would require rejection at approximately 0.1/10 = 0.01 for the roughly 10 environmental chemicals and 10 estrogen variables considered in our study.

## Results and discussion

100 % of subjects had detectible levels of at least five chemicals in their urine, and 74% had detectible levels of eight or more chemicals. Of note, 100% of subjects demonstrated presence of at least one chemical in each class of BPA, phthalates, and parabens. No significant differences were found in mean values between males and females or between age groups for any of the chemicals.

14 % of subjects had all ten chemicals present. Notably, 28 % of subjects had measurable levels of 4NP (Table 1). Existing research on 4NP is very limited, with a range of findings. While prior research has documented detectible 4NP levels in adults [12, 16, 20], this is the first known published study of 4NP exposure in American children. Interestingly, prior research in a general Belgian population of 131 subjects aged 1 to 75 (average age of 29.6) did not find 4NP in detectible levels in the urine samples analyzed [15]. Another study in 11 human tissue samples of patients aged between 9 and 62 years (average age of 34 years), found mostly undetectable levels of 4NP and concluded the chemical is not of significant importance for human exposure [17]. However, a study measuring nonylphenol in Taiwanese children aged 6.5–15.0 years found NP in 84 to 94 % of samples measured for NP concentrations of 10–250 ng/mL [19]. The wide variation of levels documented in existing research coupled with our finding that 4NP is present in nearly 1/3 of this pediatric population suggests a need for further evaluation of this ubiquitous chemical.

Analysis of the estrogen variables shows there were no significant differences by age. As there were no zero values for estrogen metabolites, all were above the limit of detection. Upon analysis by gender, females have larger maximum, standard deviations and slightly larger means for 2-OHE2 and 2-OHE1 + 2 (Table 2). 16a-OHE1 was the only estrogenic metabolite significantly different (higher, in this case) in girls than boys, with a *p* value of 0.013 from the Wilcoxon test. Elevated levels of this metabolite are linked to increased risk and worse prognosis for estrogen-sensitive cancers and autoimmune conditions [30], though the specific levels measured in this study are not directly associated with known health risks. It is not known if girls simply have relatively higher levels of 16a-OHE1 than boys.

**Table 2:** Summary of estrogen metabolites

	All subjects (*N* = 50)				25 Males				25 Females				*P* values for Wilcoxon test for males v. females
	ng/mg creatinine				ng/mg creatinine				ng/mg creatinine				
	Min	Median	IQR	Max	Min	Median	IQR	Max	Min	Median	IQR	Max	
2-16Ratio	2.50	17.45	92.05	1386.30	3.00	27.40	91.80	655.00	2.50	11.00	25.20	1386.30	0.14
4-OHE1	0.07	0.08	0.13	1.50	0.07	0.07	0.13	1.50	0.07	0.10	0.13	0.50	0.74
LOD = 0.067 ng/mL													
2-OHE1	0.15	0.15	0.23	2.40	0.15	0.15	0.25	2.10	0.15	0.15	0.15	2.40	0.60
LOD = 0.150 ng/mL													
2-OHE2	0.06	1.25	5.70	101.00	0.10	1.80	6.90	66.80	0.06	1.10	1.80	101.00	0.40
LOD = 0.065 ng/mL													
2-OHE(1 + 2)	0.30	1.70	6.35	101.20	0.40	2.00	6.70	67.50	0.30	1.70	2.30	101.20	0.72
16a-OHE1	0.07	0.07	0.03	0.50	0.07	0.07	0.00	0.20	0.07	0.10	0.13	0.50	0.013
LOD = 0.073 ng/mL													
2-OMeE1	0.10	0.10	0.10	2.10	0.10	0.10	0.10	2.10	0.10	0.10	0.00	0.40	0.37
LOD = 0.103 ng/mL													
4-OMeE1	0.23	0.23	0.00	2.10	0.23	0.23	0.00	2.10	0.23	0.23	0.00	0.40	0.60
LOD = 0.230 ng/mL													

Note: Full names of the abbreviated estrogen metabolites and ratio are in the List of Abbreviations

Since the data is skewed and non-normal, we use the Spearman’s correlation coefficient (*ρ*) to assess the correlations between estrogen markers and chemicals. Using a test to assess whether *ρ* is zero or not [31], we found that none of the correlations were statistically significant at the 0.1 level after accounting for multiple testing.

There was a widely divergent single outlier identified for most chemicals: the 22^nd^ male had high values of triclosan, 21^st^ male of 4NP and the 23^rd^ male of MetP. The 23^rd^ female had high values of 4NP and propylparaben, and the 24^th^ female of ethylparaben. It would be interesting to explore the possibility of subpopulations that are particularly at risk of higher exposures to these chemicals and/or increased risk for health effects from exposures. But with only 3 such subjects we are not able to do this for our data. Therefore, this pilot study suggests the need for additional research with a larger population.

### Limitations

A limitation of this study was the small sample size (50), with only 5 boys and 5 girls in each age group. There is an instance of an extremely large chemical outlier value. This outlier greatly affected the correlation coefficient between chemical concentrations and estrogen metabolism markers. All the other data points showed no correlation between the variables. Further investigation into the measurement process would need to be done to determine if the outlier value is spurious. It must be noted that even if the value is not spurious, an inference cannot be drawn based on one observation. The small sample size may be a reason we are unable to determine any correlation between chemical concentrations and estrogen metabolism markers. Considering the lack of statistically significant difference in estrogen metabolism between boys and girls in this study, it may be that pre-pubertal children do not demonstrate this correlation while post-pubertal adolescents and adults do, perhaps as a function of the low estrogen values in pre-pubertal children. Additionally, after pubertal onset, the associations may be sexually dimorphic, as has been observed in some pediatric cohorts [19].

Further research evaluating larger samples sizes of pre- and post-pubertal subjects would be of value, given the presence of eight or more hormonally disruptive chemicals in nearly three-fourths of subjects.

## Conclusions

Endocrine disrupting environmental chemicals were detected in all children in the study, and nearly three-quarters of subjects had detectible levels of eight or more chemicals. Nearly 1/3 of subjects had measurable levels of 4NP in their urine, a novel finding, suggesting a need for more research regarding the presence and impact of this chemical. After the removal of outliers, no associations were found between the urine levels of chemicals tested and estrogen metabolites. However, the presence of multiple chemicals in a majority of children’s urine coupled with increasing prevalence of pediatric hormonal disorders warrants further research to elucidate potential causal mechanisms in pre- and post-pubertal children.

## Abbreviations

APs: alkylphenols
APEs: alkylphenolethoxylates
BPA: Bisphenol A
DIEHC: The Deirdre Imus Environmental Health Center®
4NP: 4-Nonylphenol
DEHP: Di-2-ethylhexyl phthalate
MEHHP: Mono-(2-ethyl-5-hydroxyhexyl) phthalate
MEHP: Mono-2-ethylhexyl phthalate
MEOHP: Mono-(2-ethyl-5-oxohexyl) phthalate
MEtP: Monoethyl phthalate
2-OHE1: 2-Hydroxyestrone
2-OHE2: 2-Hydroxyestradiol
16a-OHE1: 16a-Hydroxyestrone
4-OHE1: 4 Hydroxyestrone
2-OMeE1: 2-Methoxyestrone
4-OMeE1: 4-Methoxyestrone
