# Erratum to: Association of environmental chemicals & estrogen metabolites in children

**DOI:** 10.1186/s12902-016-0085-y

**Published:** 2016-01-27

**Authors:** Erin Speiser Ihde, Ji Meng Loh, Lawrence Rosen

**Affiliations:** The Deirdre Imus Environmental Health Center®, Hackensack University Medical Center, 30 Prospect Ave, Research Building, Hackensack, NJ 07601 USA; Department of Mathematical Sciences, NJ Institute of Technology, University Heights, Newark, NJ 07102 USA

Erratum to: BMC Endocrine Disorders DOI 10.1186/s12902-015-0079-1

The original version of this article in *BMC Endocrine Disorders* [1] contained the following errors:

- The first sentence of the results section in the abstract incorrectly stated the following sentence:

100 % of subjects had detectible levels of at least one chemical in their urine, and 74 % had detectible levels of eight or more chemicals.

The corrected sentence should read:

100 % of subjects had detectible levels of at least five chemicals in their urine, and 74 % had detectible levels of eight or more chemicals.

- The citation of table 1 was misplaced in the text. Find the correct placement of the table citation below:

No significant differences were found in mean values between males and females or between age groups for any of the chemicals (Table 1).

The original article has been updated with the changes.
